# Mechanical Characterization of Graphene—Hexagonal Boron Nitride-Based Kevlar–Carbon Hybrid Fabric Nanocomposites

**DOI:** 10.3390/polym14132559

**Published:** 2022-06-23

**Authors:** Srivatsava Madarvoni, Rama P. S. Sreekanth

**Affiliations:** School of Mechanical Engineering, VIT-AP University, Amaravati 522337, Andhra Pradesh, India; msv354@gmail.com

**Keywords:** FRPs, hybrid fabric, mechanical properties, simulation, dynamic viscosity

## Abstract

Polymer nanocomposites have been gaining attention in recent years. The addition of a low content of nanomaterials into the matrix improves mechanical, wear, thermal, electrical, and flame-retardant properties. The present work aimed to investigate the effect of graphene and hexagonal boron nitride nanoparticles on Kevlar and hybrid fiber-reinforced composites (FRP). Composites are fabricated with different filler concentrations of 0, 0.1, 0.3, and 0.5 wt.% by using a hand layup process. Characterizations like tensile, flexural, hardness, and impact strength were evaluated separately, heat deflection and viscosity properties of the epoxy composites. The dynamic viscosity findings indicated that a higher concentration of filler material resulted in nano-particle agglomeration. Graphene filler showed superior properties when compared to hexagonal boron nitride filler. Graphene showed optimum mechanical properties at 0.3 wt.%, whereas the hBN filler showed optimum properties at 0.5 wt.%. As compared to Kevlar composites, hybrid (carbon–Kevlar) composites significantly improved properties. As compared to graphene-filled composites, hexagonal boron nitride-filled composites increased scratch resistance. Digimat simulations were performed to validate experimental results, and it was observed that hybrid fabric composites exhibited better results when compared to Kevlar composites. The error percentage of all composites are within 10%, and it was concluded that hybrid–graphene fiber composites exhibited superior properties compared to Kevlar composites.

## 1. Introduction

Recent scientific advances in composites have opened the way for fiber-reinforced composites (FRP) for lightweight and imperishable structural applications like aerospace, automotive, rocket, and sports, which demand multifunctional properties. To achieve these multifunctional properties, nanoparticles are being added to polymer matrix systems. Several types of particles, such as silica, carbon nanotube (CNT), and clay have been added to FRPs. These nanoparticles enhance good mechanical properties but due to an insulating property of epoxy, thermal properties are poor in FRP composites [1]. To address this issue, in recent years researchers have been exploring nanomaterials that can exhibit good mechanical as well as electrical properties. Hexagonal boron nitride (hBN) has two-dimensional SP^2^ honeycomb structures similar to graphene, due to which these two materials have excellent mechanical and thermal properties, which have recently attracted a lot of interest. Graphite, hexagonal boron nitride (hBN), and graphene nanomaterials are most suited for improving the mechanical and electrical properties of epoxy composites [2].

Sharma et al. [3] investigated the mechanical properties of multiwalled carbon nanotube Kevlar fabric composites. They observed properties at 0.3 wt.%, showing a maximum tensile strength of 93%; a 56% improvement in tensile strength and Young’s modulus compared to pristine Kevlar. Ahmade et al. [4] investigated the impact and hardness properties of graphene Kevlar composites and found 0.2 wt.% graphene, showing maximum impact and hardness values of 1.6 joules and 82, respectively. Hallad et al. [5] investigated the mechanical properties of Kevlar composites and reported that tensile strength was increased by 117% at 0.3 wt.% of graphene compared to pristine Kevlar. They also found that flexural strength was increased by 48%. From TGA, they observed that up to 330 °C the samples were thermally stable. Naveen et al. [6] investigated the effect of graphene on the ballistic performance of a hybrid (Kevlar and Cocos nucifera) composite and found that at 0.25 wt.%, filler energy absorption increased by 8.5% in a nine-layered composite and by a 12% increment in twelve-layered composites. It was also observed that at 0.5 wt.%, better interfacial bonding was displayed. Rahman et al. [7] investigated the effects of graphene and nano clay on the impact strength of Kevlar epoxy composites and reported that 10 wt.% of nano clay improved 26% of impact strength compared to pristine Kevlar composites. Graphene resulted in no improvement in impact strength. The addition of graphene and nano clay improved damage resistance to UV and water absorption. Truong et al. [8] investigated the effects of different fillers on carbon fiber composites at elevated temperatures and found short multiwalled carbon nanotubes functionalized COOH filler, showing a maximum improvement of 20.7%, 45.7%, and 73.8% in tensile strength, ultimate strain, and toughness values, respectively, and also found that at 100 °C the tensile strength, ultimate strain, and toughness were decreased by 36.5%, 37.1%, and 60%, respectively. Tominaga et al. [9] investigated the influence of exfoliated hexagonal boron nitride particles on mechanical properties of composites and found that specific strength was increased by 22% and specific rigidity was increased by 37%. Zhang et al. [10] investigated the effect of boron nitride on the thermal proprieties of epoxy and found that 40 wt.% boron nitride composite showed a 76% improvement in thermal conductivity and also that the addition of 5–10 wt.% boron nitride improved glass transition temperature. Pasare et al. [11] investigated the mechanical and tribological properties of hBN-filled carbon composites and found a 43.1% increase in tensile modulus. They also found that increasing filler content reduced the specific wear rate. Ulus et al. [12] investigated the effect of seawater on basalt graphene epoxy composites by using dynamic mechanical analysis and discovered that 0.5 wt.% graphene improved glass transition temperature by 107 °C. Wazalwar et al. [13] investigated the mechanical properties and curing behavior of graphene oxide-incorporated epoxy composites and discovered an improvement of 26% in fracture energy. Nebe et al. [14] investigated the flexural properties of composites through experimental and numerical investigations and discovered that deflection improved by 74%. Bao et al. [15] investigated the tribological behavior of epoxy composites and discovered that 0.2 wt.% graphene composites exhibited better wear properties. Raci Aydin et al. [16] investigated the effect of dynamic mechanical analysis of carbon–glass hybrid laminates in different ply angles and found that 12 layered 0°/90° angle piles were best suited for natural frequencies, while 30°/60° laminates were best suited for damping. Vedanarayanan et al. [17] investigated the mechanical properties of Kevlar and ramie fiber composites and found that interlaminar shear strength, tensile, and flexural strength increased by 30%. Yadav et al. [18] investigated the effect of nano SiO_2_ on the mechanical properties of Kevlar composites and found an increase in hardness and flexural properties by 29% and 50% for 3 wt.% of composites. Srivatsava et al. [19] investigated the dynamic mechanical analysis of carbon, Kevlar, and hybrid fabric composites and found that storage modulus, glass transition temperature at 0.3 wt.%, graphene filler was higher for carbon fabric. They also investigated the circularity index of composites and found that the composites fabricated were homogeneous, based on the circularity index.

Based on the literature review, it can be observed that several works reported on the mechanical properties of carbon fabric with MWCNT, silica, clay, graphene, etc. Very few works reported on Kevlar and hybrid fabric composites to the best of our knowledge. The novelty of the present work lies in incorporating the hybrid Kevlar–carbon fabric as a matrix into the 2D nanofillers reinforced epoxy. To the best of authors’ knowledge, such work has not been reported elsewhere in the literature. The present work aims to study the effect of different wt.% graphene (0, 0.1, 0.3, 0.5) and hBN (0, 0.1, 0.3, 0.5) fillers on Kevlar and hybrid (carbon as a wrap and Kevlar weft) fiber on mechanical properties, dynamic viscosity, hardness, and heat deflection response. Digimat simulations were also performed and compared with the experimental results.

## 2. Materials and Methods

Bisphenol A diglycidyl ether (DGEBA), commonly named Epoxy Ly556, and Hardener Hy951 were obtained from Singhal Chemical Corporation, Delhi, India. Kevlar (200 GSM) and hybrid (aramid as warp and carbon as weft, 200 GSM) were obtained from Marktech companies Pvt. Ltd., Bangalore, Karnataka, India. Hexagonal boron nitride (h-BN) was obtained from Sisco Research Laboratories Pvt. Ltd., Hyderabad,Telangana,India. Hexagonal boron nitride (h-BN) platelets had an average particle size of 70 nm and surface area of 19.4 m^2^/g. The functionalization of h-BN was performed as described in the literature [20]. Functionalized graphene was obtained from Adnano Technologies, Majjigenahalli, Karnataka,India. GNP had a surface area of 112 m^2^/g, bulk density of 0.3 g/cc, and an average diameter of 10–15 microns with a purity greater than 98%.

### 2.1. Composite Fabrication

**Figure 1:**
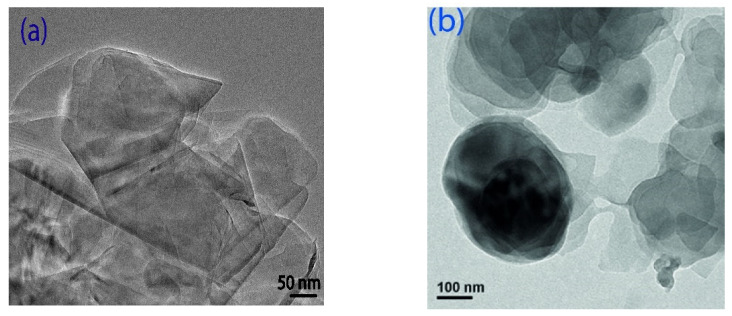

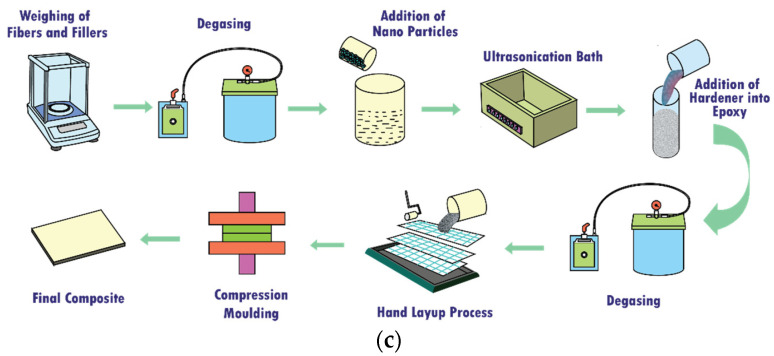
TEM images of (**a**) graphene and (**b**) hexagonal boron nitride (hBN). (**c**) Composite fabrication process.

Fabrics were washed with acetone to extract dust particles from the fiber’s surface and then dried for 24 h at room temperature. After drying, the fibers were subjected to moisture removal in a vacuum oven at 70 °C for 4 h. To ensure that no air bubbles were present in the epoxy, degassing was performed 60 min before the addition of the curing agent and 5 min after the addition of the curing agent. Graphene and hexagonal boron nitride particles at different wt.% (0, 0.1, 0.3, 0.5) were added into the epoxy individually. Ultrasonication was performed on different filler concentrations at a temperature of 80 °C for 0.5 h. Hardener HY951 was added to the epoxy with a mixing ratio of 10:1 [21]. Kevlar and hybrid composites were fabricated by using the hand layup technique, each composite consisting of nine layers of fibers. Layers were oriented in 0° and 90° directions. Fabricated composites were subjected to the compression molding process. The schematic representation of composite fabrication is shown in Figure 1c. Final composites had dimensions of 300 mm × 300 mm × 3 mm. Composites were coded according to filler concentrations listed in Table 1a,b. The TEM images of graphene and hBN fillers were shown in Figure 1a,b.

### 2.2. Tensile Testing

Tensile testing was conducted on a Universal Testing Machine (Tec-sol, Chennai, Tamil Nadu, India), according to ASTM D 3039 standard, at a crosshead speed of 2 mm/min at room temperature. The test samples were cut by using a Bosch Jigsaw machine (Bosch, Guntur, Andhraprdesh, India). The final dimensions of the cut tensile samples were 250 mm × 25 mm × 3 mm. For accuracy and repeatability, 5 samples were tested per each filler concentration and the average values were reported.

### 2.3. Flexural Testing

The flexural test was performed on a Tinius Olsen H10KL, Noida, Uttar Pradesh, India, according to ASTM D7264, at a crosshead speed of 2 mm/min. For accuracy and repeatability, 5 samples were tested per each filler concentration and the average values were reported.

### 2.4. Micro Vickers Hardness

Hardness measured by using Micro Vickers hardness tester supplied by R.S.Scientific Kolkata, West Bengal, India. ASTM E384-17 standard was used for the measure of microhardness. Hardness was measured at a constant load of 1 kg over a dwell period of 20 s. To ensure repeatability, 5 readings were taken for each configuration and the average value was reported.

### 2.5. Viscosity Measurement

Viscosity measurements were carried out on the Rheometer (MCR 102) model from Anton Paar India limited, Bangalore, Karnataka, India. A frequency sweep was performed on the epoxy filled with nanomaterials (graphene and hBN).

### 2.6. Heat Deflection Test

The heat deflection test is a measure of the ability of a polymer to carry a load at raised temperatures. The heat deflection apparatus was supplied by S.C.Dey & Co, Kolkata, West Bengal, India. ASTM D 648 or ISO 75 standards were used for the measuring. Table 2 shows the standard sample dimensions and deflection values as per the ISO standard. The sample was placed in between two supports and the load was applied to the sample. Then the sample was dipped into silicone oil and subjected to the heat of 2 °C/minute. As the temperature increased, the sample began deflection, and when the sample reached the specified standard deflection, the temperature was recorded. The test was repeated twice, and the average value was reported.

### 2.7. Impact Test

The impact test was performed on a Digital Izod/Charpy tester supplied by R.S.Scientific, Kolkata, West Bengal, India, according to ASTM D256, with sample dimensions of 63.5 mm × 12.7 mm × 3 mm. The prepared sample was placed 2 mm away from stoppers with a 21.7 Kg weight hammer at the center of the sample.

### 2.8. Scratch Test

A scratch test was performed on a Scratch Tester (DUCOM, Bangalore, Karnataka, India), according to ASTM D7027-05. Experiments were conducted under a constant load of 150 N, a ramp 30–150 N with a velocity of 1 mm/s, and scratch length of 25 mm.

### 2.9. Digimat Software

Digimat software was used for linear and nonlinear multiscale material modeling. Digimat software enables the prediction of the constitutive behavior of heterogeneous and/or anisotropic materials such as polymer matrix composites (PMC), rubber matrix composites (RMC), metal matrix composites (MMC), or even nanocomposites. It consists of different modules, namely, MF, FE, MX, MAP, CAE, RP, VA, HC, and AM. In the present study, MF and FE modules were used. MF indicates mean-field homogenization, and it uses Eshelby-based semi-analytical mean-field homogenization approaches and an analytical description of the material in order to compute the thermo-mechanical, thermal, or electrical properties of a composite as a function of its microstructure morphology, i.e., the inclusion of shape, orientation, volume/mass fraction, and micro (i.e., per-phase) material behavior. Mori–Tanaka homogenization models were used in the present study. In this uniform model, fiber distribution assumption was taken into consideration to approach the problem within the micromechanical theory of periodically arranged heterogeneous materials. The Digimat MF module was used for the evaluation of fiber volume fraction, and Young’s modulus and Poisson’s ratio of Kevlar- and hybrid fiber-reinforced graphene filler composites were calculated.

**Figure 2:**
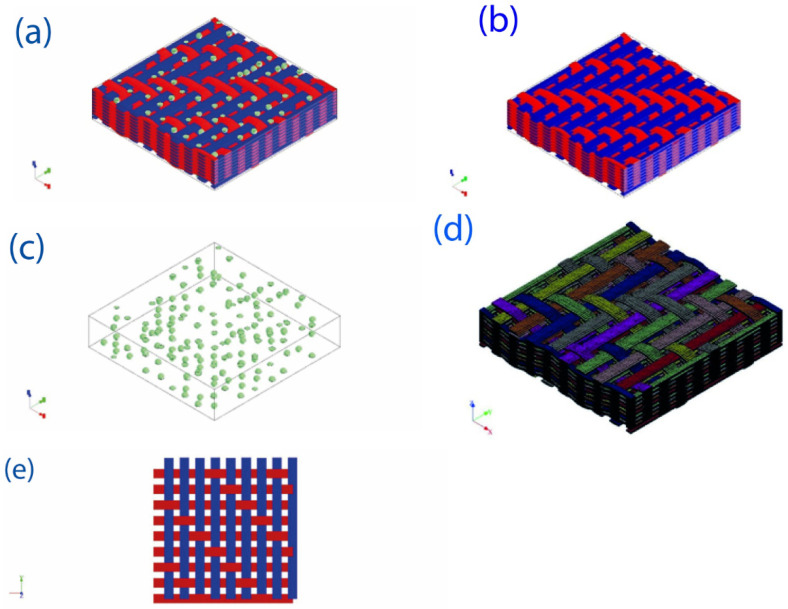

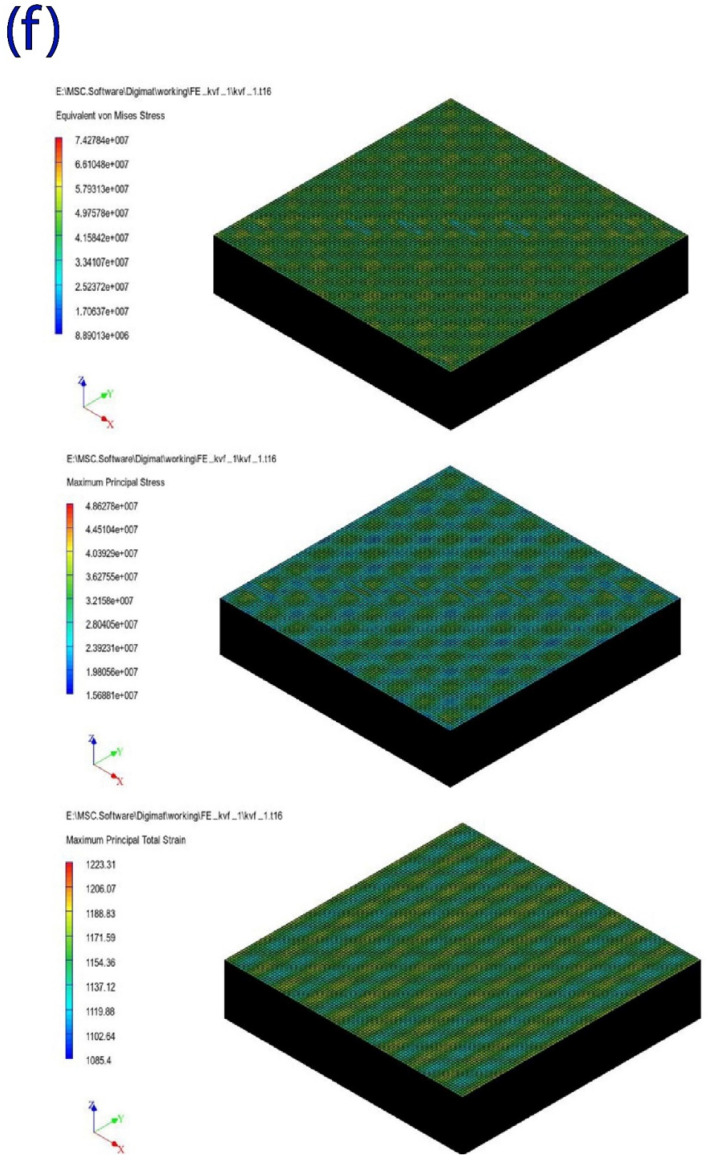
Digimat modeling: (**a**) RVE of filler and fibers matrix, (**b**) RVE of matrix and fibers, (**c**) RVE of filler, (**d**) meshing of RVE, (**e**) fabric weaving style, (**f**) Digimat simulation results.

The Digimat FE module was used for the visualization of RVE geometry and the evaluation the stress–strain analysis. Fabric microstructure was generated by 3D woven interlock wrap weft phases considered for Kevlar composites and for hybrid composites with Kevlar as a wrap and hybrid fabric (carbon–Kevlar) as weft phase, with parameters shown in Table 3 and Table 4. The fabric weaving pattern is shown in Figure 2e and the generated fabric has a unit cell of 9 mm × 9 mm × 1.9 mm. The RVE model of Fabric and filler is shown in Figure 2.

The generated RVE model was subjected to meshing (voxel) with a size of 100 mm, followed by uniaxial loading with 90° theta.

## 3. Results & Discussion

### 3.1. Tensile Strength

**Figure 3:**
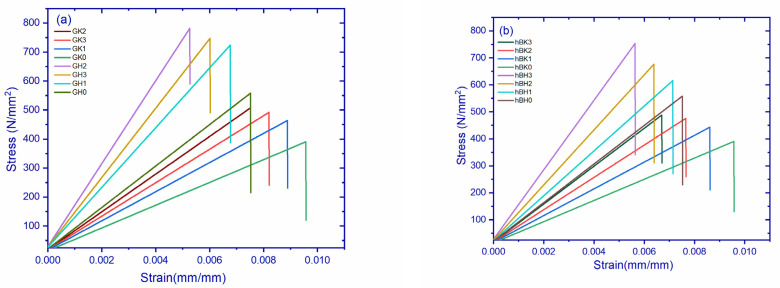
(**a**). Stress–strain curves of graphene filled composites. (**b**) Stress–strain curves of hBN-filled composites.

Figure 3a shows the stress–strain curves of GK and GH composites. It can be observed that the materials followed a linear pattern, which indicates that the composites are brittle in nature. The brittleness increased with filler content. GH composites exhibited a more brittle nature as compared to GK composites. GH2 showed a maximum stress value of 780 N/mm^2^ compared to an all-composite variation, whereas GK0 demonstrated the least value of 390.10 N/mm^2^. The reason for the superior properties of GH composites is attributed to their better dispersibility and excellent load-carrying capacity [22]. The increasing stress sequence of all composites is as follows: GH2 > GH3 > GH1 > GK2 > GK3 > GK1 > GK0.

Figure 3b shows the stress–strain curves of hBK and hBH composites. It is observed that the materials followed a linear pattern which indicates that the composites are brittle in nature. The addition of filler materials further increased the brittleness of the composites. HBH composites exhibited a more brittle nature compared to hBK composites. hBH3 showed a maximum value of 812.08 N/mm^2^, while hBK0 demonstrated the least value of stress. The stress behavior of different composites in incremental order are as follows: hBH3 > hBH2 > hBH0 > hBK3 > hBK2 > hBK1 > hBK0. The tensile properties of different composites are shown in Table 5a.

**Figure 4:**
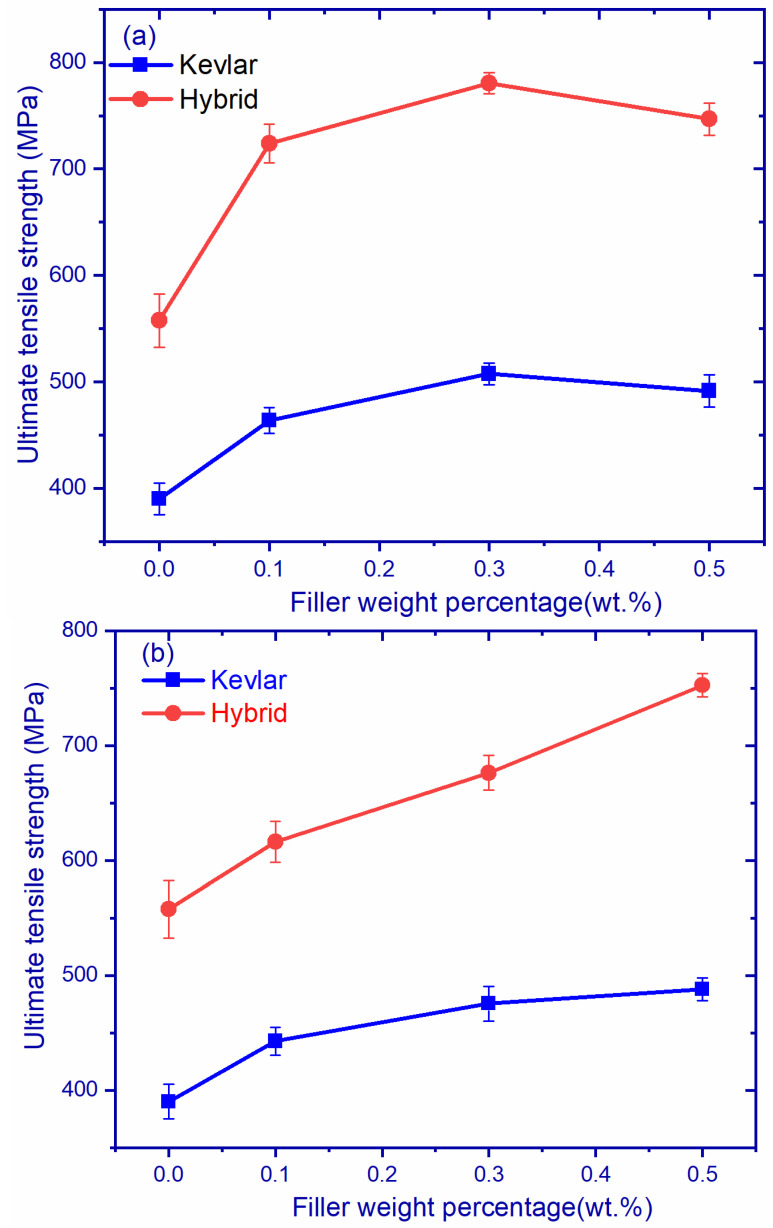

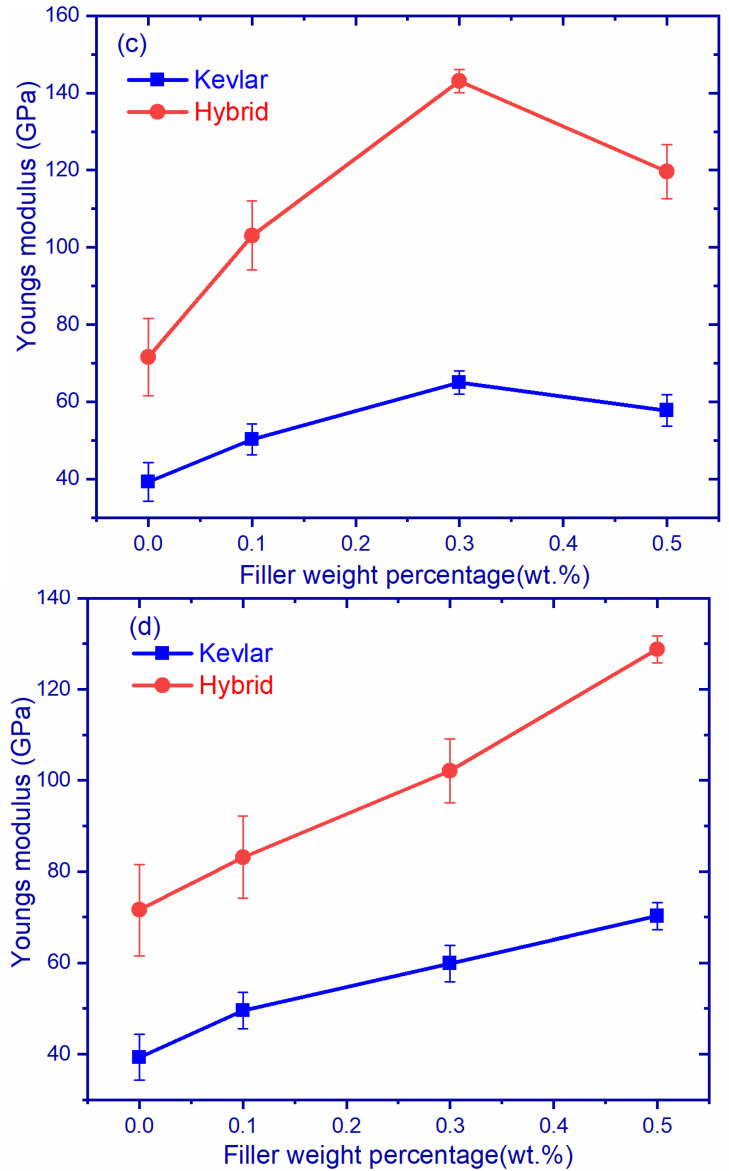
Ultimate tensile strength of (**a**) graphene-reinforced composites, (**b**) hBN-reinforced composites, Young’s modulus of (**c**) graphene-reinforced composites, and (**d**) hBN-reinforced composites.

The ultimate tensile strength comparison of GK and GH composites are shown in Figure 4a. The figure indicates that hybrid fiber composites exhibited superior strength compared to Kevlar composites. Additionally, the increase in filler content increased the strength of the composites up to 0.3 wt.% in both GH and GK composites, beyond which the strength was decreased. Increments of 29.80%, 39.97%, and 33.9% for GH1, GH2, and GH3 composites, as compared to GH0 composites, were observed and, similarly, increments of 18.86%, 30.06%, and 26.0% for GK1, GK2, and GK3, as compared to GK0, were observed, respectively. The ultimate tensile strength of 780 N/mm^2^ was observed in the GH2 composite among all composite configurations.

Young’s modulus comparison of GK and GH composites are shown in Figure 4c. it was observed that GH composites exhibited a superior Young’s modulus compared to GK composites. GH2 had the highest Young’s modulus of 89.09 GPa, and GK0 had the lowest Young’s modulus of 39.31 GPa. The Young’s moduli of different composites are shown in Table 5b. The reason for the superior Young’s modulus in GH composites is the presence of carbon fiber, enabling better bonding energy between carbon atoms of the nanomaterial.

Figure 4b,d shows the comparison of Young’s modulus and the UTS of hBK and hBH composites. It is evident that hBH composites exhibit higher values as compared to hBK and hBH3, showing a 15% improvement in Young’s modulus, i.e., 85.14 GPa. Graphs give a clear indication of improvement with the addition of hBN filler. The increase is attributed to the high modulus of hBN filler (400 to 900 GPa) [23]. It was observed that the graphs are linear, irrespective of materials and filler content. This could be due to the layup sequence (0°, 45°, 90°) of composites during fabrication [24,25] as well as the weaving pattern [26].

**Figure 5:**
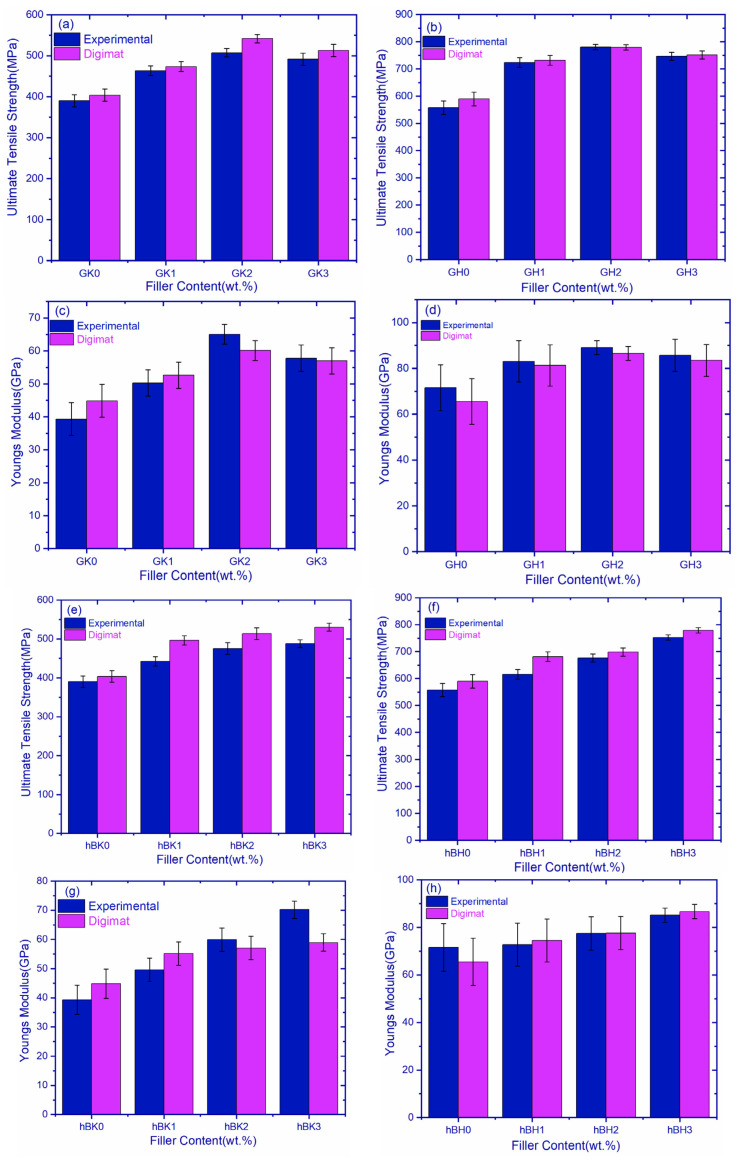
Comparison of experimental Digimat graphene-reinforced composite system—(**a**) UTS of Kevlar composites, (**b**) UTS of hybrid composites, (**c**) Young’s modulus of Kevlar composites, (**d**) Young’s modulus of hybrid composites; and comparison of experimental Digimat hBN-reinforced composite systems—(**e**) UTS of Kevlar composites, (**f**) UTS of hybrid composites, (**g**) Young’s modulus of Kevlar composites, (**h**) Young’s modulus of hybrid composites.

RVE analysis was performed using Digmat software, and the findings were compared with the experimental data, as shown in Figure 5. The comparison of the UTS of the experimental and Digimat values of GK composites is shown in Figure 5a. Table 5b shows the error percentage between the experimental and Digimat values, which ranges from 2 to 6 percent and is within acceptable bounds.

Figure 5b shows a comparison of the UTS of GH composites produced physically and via Digimat modeling. Table 5a shows the percentage difference between experimental and simulation results. The percentage variation is in the 0–5% range, which is within acceptable bounds. The experimental values of GH composites are almost identical to Digimat values in the case of GH2 and GH3 composites, with an error rate of less than 0.6%. Figure 5c,d represents a comparison of Young’s modulus of Kevlar and hybrid fiber composites incorporated with graphene filler.

Young’s modulus of Kevlar and hybrid composites incorporated with graphene are shown in Figure 5c,d. The graphs revealed that the Young’s modulus increased up to the GH2 and hBH2 composition, beyond which the composites exhibited a sightly decreased modulus. The variation in simulation results and experimental results observed in the graphs may be due to many factors, such as the composite fabrication, weather, and mixing of nanoparticles during experimentation.

The ultimate tensile strength of hBK composites obtained experimentally and those obtained using Digimat is shown in Figure 5e. Table 5a displays the error percent of experimental and Digimat results. The error percentage of values ranges between 2 and 10%, which is within acceptable bounds. hBK2 has the lowest mistake rate of 7.4%. Figure 5f depicts a comparison of the ultimate tensile strength of hBH composites from experimental and Digimat simulations. Table 5a shows the error percentages of experimental and Digimat values, which vary from 3 to 10% and are within acceptable ranges; nevertheless, hBH2 has the lowest error of 3.2%.

Young’s modulus of Kevlar and hybrid composites incorporated with graphene are shown in Figure 5g,h. It is observed that the Young’s modulus increased up to hBK3 and hBH3. Simulation results revealed that hybrid composites are more accurate when compared with experimental results.

From the above discussion, it can be observed that 0.3 wt.% graphene exhibits superior UTS and Young’s modulus in all combinations; this may be due to the uniform dispersion of particles through the fiber surface and epoxy. Uniform dispersion ensures better interlocking between the fiber and matrix parts, which results in the improvement of the load-carrying capacity of the composite. At 0.5 wt.%, the UTS and Young’s modulus decreased because of the agglomeration caused by Van der Waals forces [27]. An increase in filler content increases the bonding surface area, which in turn results in a decrease in bonding strength [28]. Inadequate bonding strength leads to reduced loads transferred from the matrix to the fiber, which also results in crack propagation.

RVE FE analysis of hybrid composites is shown in Figure 2f. It can be observed from the diagram that the equivalent von Mises stress, maximum principal stress, and the maximum total strain of the composite are within the acceptable limits, which indicates that the applied load on the composite was absorbed by the composite without any deformation or failure.

### 3.2. Dynamic Viscosity

**Figure 6:**
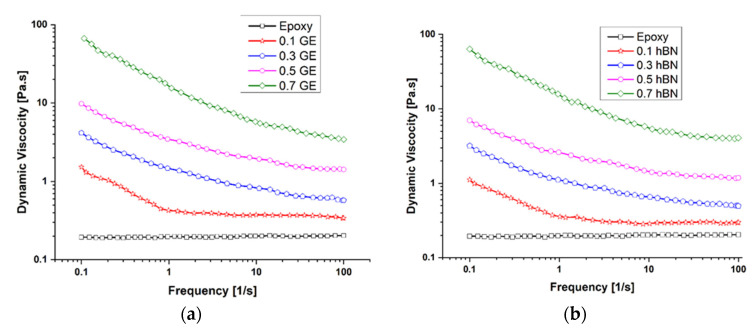
Dynamic viscosity of epoxy with (**a**) graphene and (**b**) hBN fillers at different loadings.

Viscoelastic materials behave linearly up to the critical strain level, beyond which the materials behave non-linearly. Therefore, a strain sweep was initially performed to estimate the linear viscoelasticity of the epoxy-filled graphene and hBN composites. The dynamic viscosity of epoxy filled with graphene and hBN is shown in Figure 6a,b. The addition of fillers increased the storage modulus, which led to an increase in the viscosity of composites.

The dynamic viscosity of pure epoxy incorporated with graphene and HBN filler was observed to decrease as the angular frequency increased. The behavior of neat epoxy is similar to a Newtonian fluid at lower frequencies, but as the frequency increases, it shows a shear-thinning effect. The addition of nanofiller content linearly increased the viscosity of epoxy. At higher viscosity, it is difficult to mix the nanofillers properly into the epoxy matrix, which leads to the agglomeration of nanoparticles, leading to the poor strength of the matrix. Another problem at higher viscosity is that while carrying out the hand layup process it becomes exceedingly difficult to roll epoxy smoothly over the entire composite laminate. Up to 0.5 wt.% filler content, the viscosity of filler increases proportionately, whereas at 0.7 wt.%, the jump in the viscosity is high, which leads to a higher chance of agglomeration at 0.7 wt.%. Therefore, in the present study, we experimented with up to 0.5 wt.% of filler content.

### 3.3. Flexural Test

**Figure 7:**
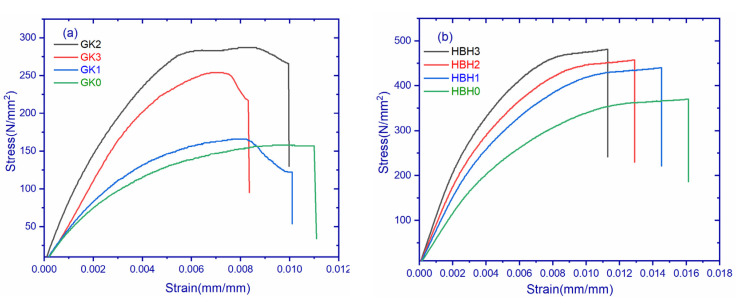

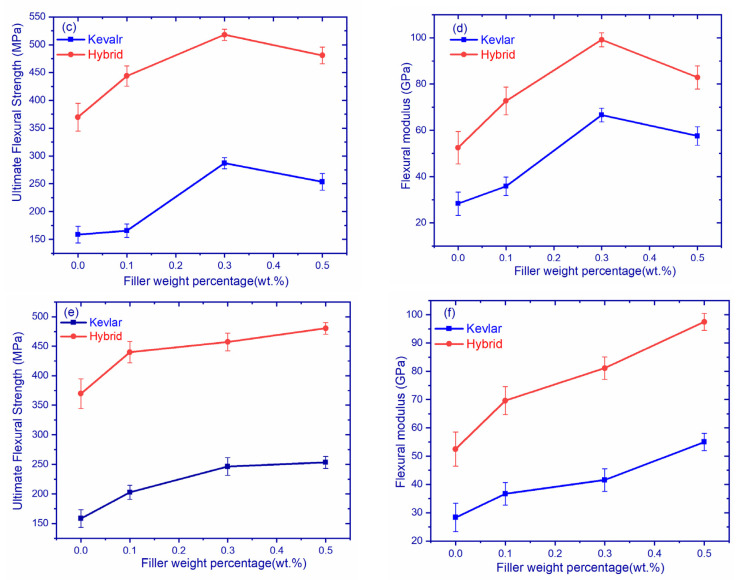
Flexure response of (**a**) graphene–Kevlar, (**b**) hBN–Kevlar; ultimate flexural strength of (**c**) graphene system, (**d**) hBN system; Flexure modulus of (**e**) graphene system, (**f**) hBN system.

Figure 7a illustrates the stress–strain curve of graphene–Kevlar composites. The figure indicates that the addition of filler content increased the stress of composites, with GK2 showing the maximum value of 287 N/mm^2^. It should be noted that beyond GK2, the addition of filler content resulted in decreased values. All composites exhibited no delamination until peak stress but thereafter displayed a significant drop due to crack propagation inside the composites, beyond which the composite continued to bear load but never achieved peak values. Figure 7c,e shows the comparison of Kevlar- and hybrid fabric-based graphene composites with different filler contents. The values of flexural strength and modulus are listed in Table 6a. It should be noted that an increment of 38.5%, 88.91%, and 57.91% of GH1, GH2, and GH3 was seen in the flexural modulus of the hybrid–graphene composite. Maximum flexural strength was achieved by GH2 composites, i.e., 517.96 MPa, and a maximum flexural modulus, i.e., 99.6 GPa, for hybrid–graphene-based composites. The optimum loading was at 0.3 wt.% graphene, beyond which the property decreased due to the rapid aggregation of graphene sheets, which behaved like micro fillers with less surface area. These agglomerates behave as an obstacle [29] in polymer flow, which results in voids being created [30] between epoxy and fiber. Due to these voids, when a load is applied on to composites there is a chance of zero load transfer to fibers. In fiber-reinforced composites, fibers act as high load carriers. However, in the current scenario, due to the non-transfer of load, epoxy cannot carry the load and therefore results in crack propagation and ultimately in the failure of the composite.

Figure 7b illustrates the stress–strain response of hBN–Kevlar composites. It should be noted that the addition of hBN content increased the flexural modulus and flexural strength, as shown in Table 6b. A comparison of flexural strength and modulus of different materials is shown in Figure 7e,f. It can be observed from the graphs that the hybrid-based composites have a maximum flexural strength and maximum flexural modulus. The addition of hBN filler improved 30% of flexural strength in the hBH3 composite, whereas an 85% of improvement in the flexural modulus of the hBH3 composite was observed. A high modulus indicates that the material is tougher [31] and the addition of filler content improved flexural strength and modulus due to the uniform dispersion of particles [32], resulting in good bonding strength between the fiber and matrix. Additionally, due to the large surface area of hBN particles, contact with the matrix enables load transfer to the matrix and fibers [33].

### 3.4. Hardness

**Figure 8:**
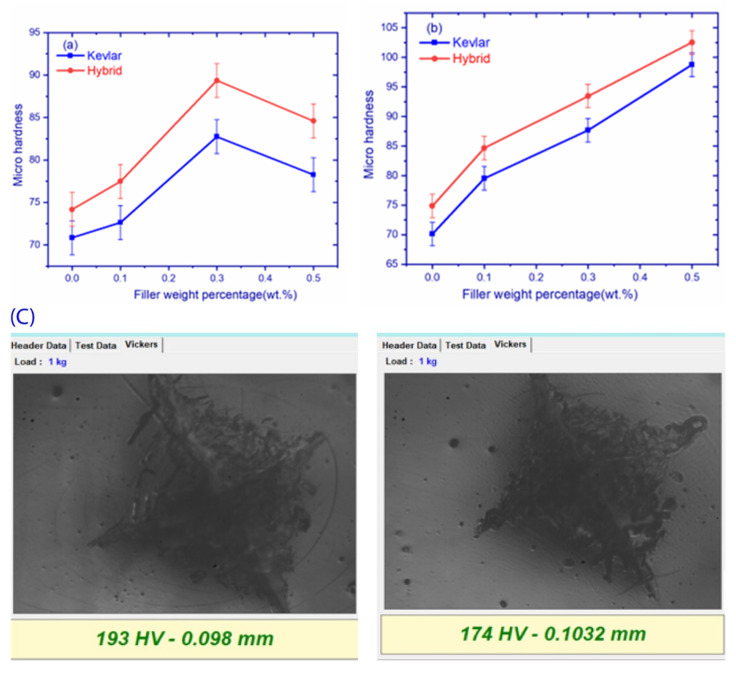

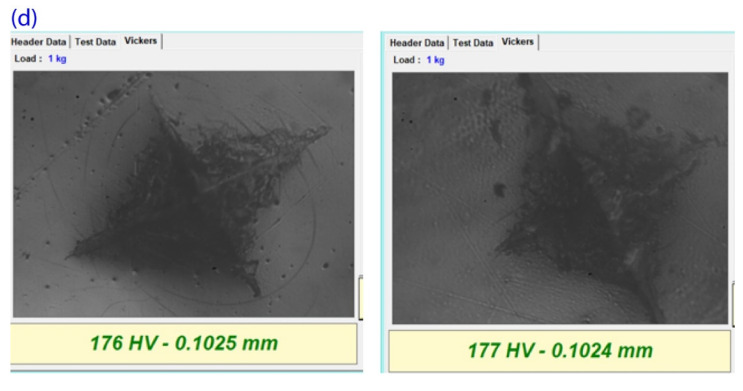
Micro-Vickers hardness of (**a**) graphene composites, (**b**) hBN composites, and micro-Vicker hardness images of (**c**) graphene composites, (**d**) hBN composites.

The hardness values of graphene-based fiber composites are depicted in Figure 8a. Kevlar composites exhibited lower hardness, whereas carbon composites showed high hardness. The highest values of hardness were observed in GK2 composites at 200 HV and the least at 159 HV in the GK0 composite. An increment of 38.36% and 12.20% in hardness of Kevlar and hybrid composites can be observed, and the hardness values are shown in Table 7. From the figure, it can be noted that the addition of filler increased hardness up to an optimum value, beyond which a minor drop in hardness values in all composites was seen. A direct relationship between stiffness and modulus was established by [31], where the results were presented, following the criteria, and maximum modulus was observed at 0.3 wt.%. Indent images of graphene and hBN composites are shown in Figure 8c,d.

Figure 8b illustrates a hardness comparison of hBN-filled Kevlar and hybrid composites. The maximum hardness exhibited by a hBK3 composite, i.e., 200 Hv. Hardness values of all composites are shown in Table 8. The addition of hBN filler content increased hardness due to the high hardness of the hBN filler compared to epoxy. When a high hardness filler is incorporated into the epoxy, the overall hardness of composite also increases [34]. The addition of hBN filler to increase hardness was also reported by Alqahtani et al. [35].

### 3.5. Izod Impact Test

**Figure 9:**
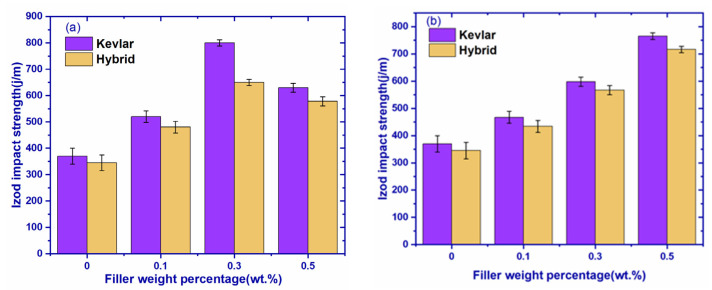
Izod impact strength of (**a**) graphene composites and (**b**) hBN composites.

The impact test was performed according to ASTM D 256. The impact strength of various graphene-based composites was shown in Figure 9a. The impact strength of graphene-filled composites is shown in Table 8. Figures indicates superior impact properties for Kevlar fiber composites when compared to hybrid fiber composites. It was observed that 0.3 wt.% of graphene filler showed maximum impact strength in all-fiber composites. The GK2 composite exhibited maximum impact strength of 800 J/m, which was 116.2% higher than the Kevlar composite without graphene. The impact strength of different graphene composites in sequential order is as follows: GK2 > GH2 > GK3 > GH3 > GK1 > GH1 > GK0 > GH0.

The impact strength of hBN-filled composites is shown in Figure 9b and tabulated in Table 8. The figure indicates superior impact properties for Kevlar fiber composites when compared to hybrid fiber composites. It can be observed the addition of hBN filler increased the impact strength of the composites. The maximum impact strength was observed at hBK3 composites, i.e., 765 J/m. The addition of hBN filler resulted in a 51%, 38%, and 26% increase in GK3, GK2, and GK1 composites, whereas a 51%, 39%, and 20% increase was observed in GH3, GH2, and GH1 composites, respectively, as compared to pure samples. Graphene-filled composites exhibited slightly higher impact strength compared to hBN-filled composites until optimum filler concentration.

### 3.6. Heat Deflection Test

**Figure 10:**
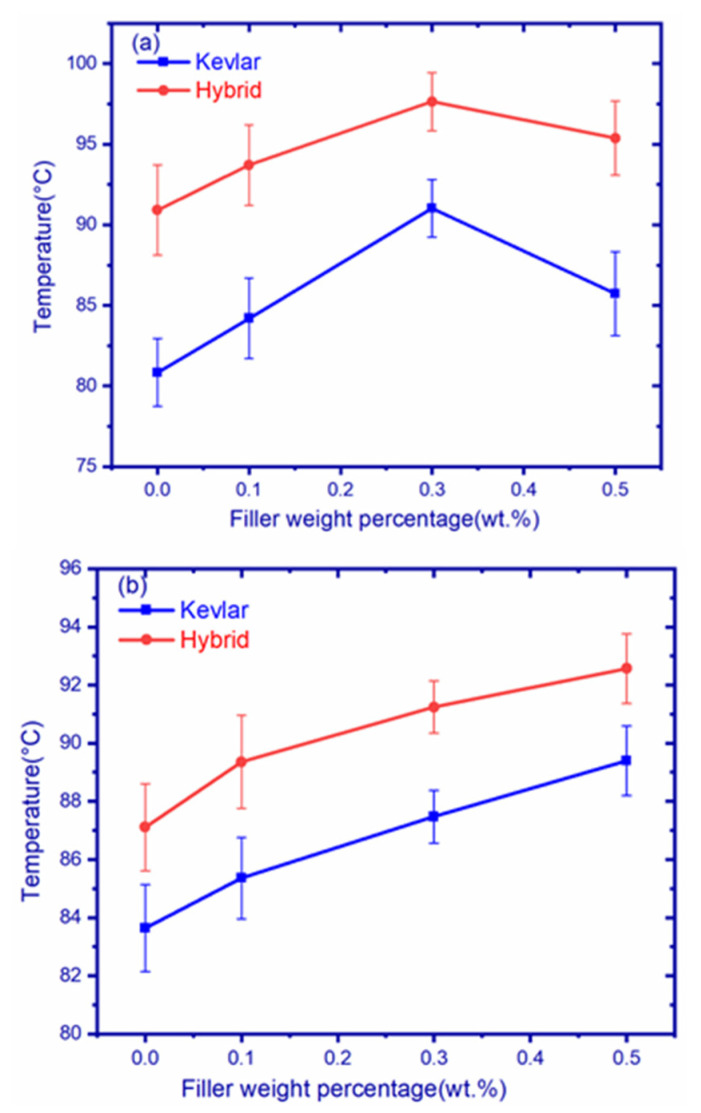
Heat deflection temperature of (**a**) graphene composites and (**b**) hBN composites.

Heat deflection curves of graphene-filled composites are shown in Figure 10a, and the heat deflection temperatures of different composites are listed in Table 9. From the figures it can be observed that the hybrid–graphene fiber composites exhibited superior temperature compared to Kevlar–graphene fiber composites. The addition of graphene filler improved temperatures up to GK2, and GH2 composites thereafter decreased. GH2 composites showed a maximum temperature of 97.65 °C. The heat deflection temperature of different composites is shown in the following sequential order: GH2 > GH3 > GH1 > GK2 > GH0 > GK3 > GK1 > GK0.

Heat deflection curves of hBN-filled composites are shown in Figure 10b, and the heat deflection temperatures of different composites are listed in Table 9. The figure indicates that hybrid–hBN fiber composites exhibited superior temperatures compared to Kevlar–hBN composites. The addition of hBN filler resulted in the continuous improvement of temperature in composites. hBH3 composites showed a maximum temperature of 92.57 °C. The addition hBN filler resulted in an increase of 5.8%, 4.5%, and 2.5% in hBH3, hBH2, and hBH1 composites but an increase of 9.7%, 7.8%, and 5.5% in hBK3, hBK2, and hBK1 composites, respectively, compared to pure samples.

### 3.7. Scratch Test

**Figure 11:**
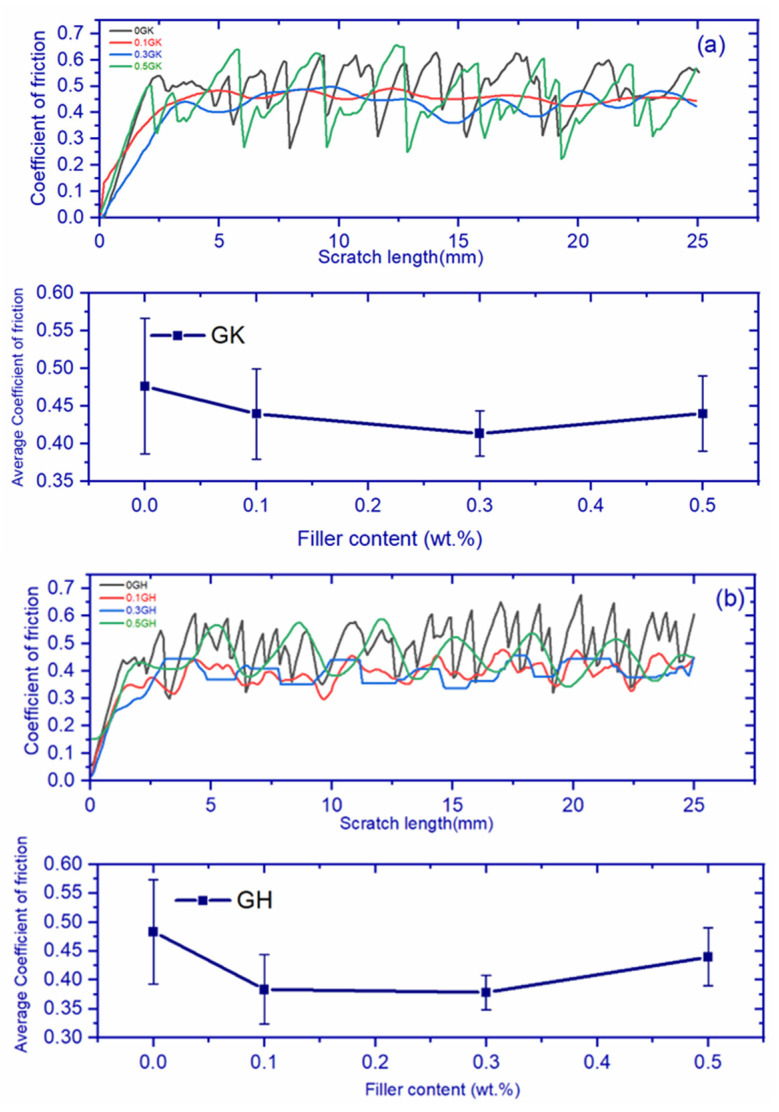

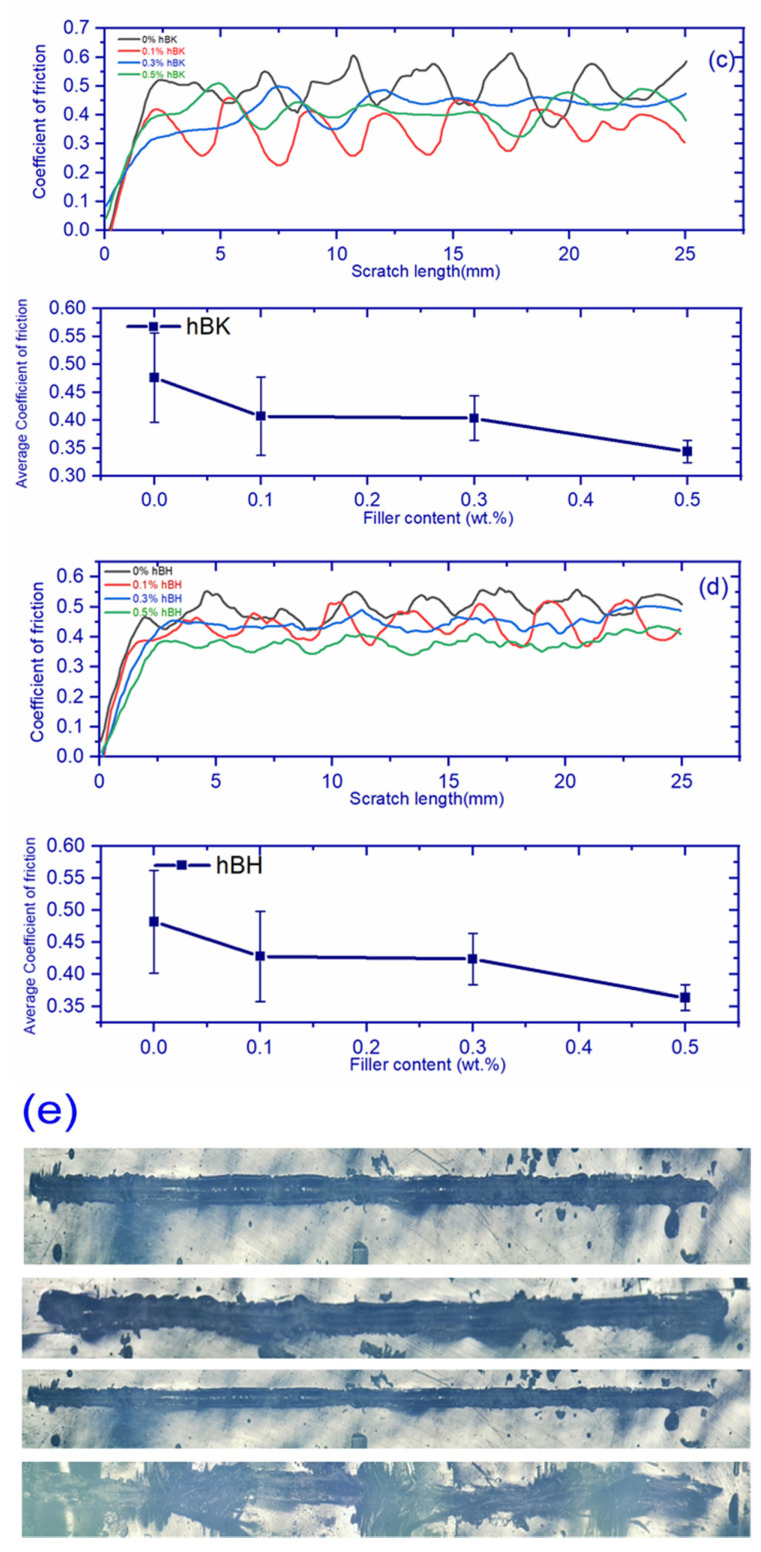
Scratch test of (**a**,**b**) graphene composites and (**c**,**d**) hBN composites; (**e**) sample scratch image of graphene composites.

Figure 11a,b illustrates the relationship between the coefficient of friction and the scratch length of the graphene-based hybrid fiber and Kevlar fiber composite system. From the figure it can be observed that there is no influence of the scratch length on the coefficient of friction, but the coefficient of friction varies from 0.3 to 0.6 as the indenter moves across the surface of the composite up to the defined scratch length. This is due to the fact that when the indenter applied load on the composite surface for the first time, the coefficient of friction increased linearly and initially fluctuated between certain values because of the fiber surface. When the indenter moves, it cuts the fiber’s surface, and whenever it cuts’s the fiber surface, it shows a high coefficient of friction [36]; however, a low coefficient of friction is observed for pure samples. The addition of fillers into composites resulted in the strengthening of the matrix surface, and thereby we can observe less fluctuation of the coefficient of friction. From the figures, a similar kind of mechanism can be observed in the HBN-based filler composites shown in Figure 11c,d. Figures showed fluctuations in the reading and therefore the average coefficient friction was considered. Graphene-based filler composites showed an optimum friction coefficient at 0.3 wt.%, whereas boron nitride filler displayed 0.5 wt.% as the optimum concentration for the friction coefficient. The addition of fillers resulted in noise reduction in the coefficient of friction of the materials. The variation of load increased the scratch width and the difficulty of capturing the full-width image through the image acquisition system, which resulted in the scratches not looking straight. Some chipping was observed on the scratch line, which indicates that the applied load is higher and looks like a dent on the surface of the composite. The scratch image (Figure 11e) reveals that the scratch looks like a plowing mechanism [37] and adhesive failure occurred during the scratch test. The addition of nanomaterials increased adhesion [38] between the fibers and matrix, which resulted in good interfacial bonding. Composites that have good interfacial bonding increase in resistance to scratch and penetration.

## 4. Conclusions

In the present research work, a comparative study on the effect of graphene and hBN fillers on carbon, Kevlar, and hybrid composites and their mechanical properties was evaluated. Based on the study, the following conclusions can be drawn:

- Hybrid composites show a higher flexural modulus, which demonstrates their usability for compressive and bending load applications. The flexural modulus in the HBH3 composite was increased by 85%.
- Hardness, tensile strength, and heat deflection of graphene-based composites exhibited superior properties when compared to HBN-based composites. The optimum properties were found at 0.3% filler content.
- Digimat simulations were close to experimental results regarding ultimate tensile strength. Simulation results suggested that the applied load conditions are within acceptable limits.
- Kevlar’s impact resistance properties were greatly enhanced by the addition of fillers and thus enhancing their applicability.

## Figures and Tables

**Table 1:** (**a**). Specification material according to GNP wt.%. (**b**) Specification of material according to h-BN wt.%.

a		
**Symbol**	**No of Layers**	**GNP (wt.%)**
GK0	9	0
GK1	9	0.1
GK2	9	0.3
GK3	9	0.5
GH0	9	0
GH1	9	0.1
GH2	9	0.3
GH3	9	0.5
**b**		
**Symbol**	**No of Layers**	**GNP (wt.%)**
hBK0	9	0
hBK1	9	0.1
hBK2	9	0.3
hBK3	9	0.5
hBH0	9	0
hBH1	9	0.1
hBH2	9	0.3
hBH3	9	0.5

**Table 2:** ASTM/ISO standard dimension of the heat deflection test.

	Load	ASTM Standard Deflection (mm)	ISO Standard Deflection (mm)		ASTM Sample Dimensions (mm)			ISO Sample Dimensions (mm)			
Method A	1.8 MPa	0.25	Flat	Edge	L	B	H		L	B	H
Method B	0.45 Mpa		0.32	0.34	50.8	12.6	6.35	Flat	80	10	4
								Edge	120	10	4

**Table 3:** Parameters for microstructure generation of fabric.

Woven Pattern	Twill
Number of warp yarns	9
Number of weft yarns	9
Number of layers	9
Warp depth	2
Weave step	1
Warp yarn count	10
Weft yarn count	10
Yarn spacing ratio	0
Yarn crimp	0.1

**Table 4:** Fiber yarn phase properties.

Fiber Yarn	Kevlar, Hybrid
Yarn linear density	43.96 tex
Fiber diameter	0.05 mm
Fiber volume fraction	0.66
Yarn height	0.1 mm
Yarn width	0.6 mm

**Table 5:** (**a**). Tensile values of hBN-filled composites. (**b**) Tensile values of graphene-filled composites.

a							
Fabric	Filler Weight Percentage	Ultimate Tensile Strength (MPa)	Young’s Modulus (GPa)	Ultimate Tensile Strength (MPa) Digimat	Young’s Modulus (GPa) Digimat	UTS Error	Young’s Modulus Error
Kevlar	hBK0	390.10	39.31	403.66	44.85	3.3	12.3
	hBK1	442.65	49.58	496.61	55.17	10.8	10.1
	hBK2	475.44	59.90	513.73	57.08	7.4	4.7
	hBK3	488.09	70.20	530.46	58.94	7.9	16.0
Hybrid	hBH0	557.67	71.54	589.92	65.54	5.4	8.3
	hBH1	616.13	72.78	681.76	74.54	9.6	2.3
	hBH2	676.29	77.46	698.68	77.63	3.2	0.2
	hBH3	752.67	85.14	779.15	86.64	3.3	1.7
**b**							
**Fabric**	**Filler Weight Percentage**	**Ultimate Tensile Strength (MPa)**	**Young’s Modulus (GPa)**	**Ultimate Tensile Strength (MPa)** **Digimat**	**Young’s Modulus** **(GPa)** **Digimat**	**UTS Error**	**Young’s Modulus Error**
Kevlar	GK0	390.10	39.31	403.66	44.85	3.3	12.3
	GK1	463.70	50.29	473.65	52.62	2.1	4.4
	GK2	507.40	65.04	541.78	60.13	6.3	7.54
	GK3	491.55	57.78	513.10	57.01	4.1	1.3
Hybrid	GH0	557.67	71.54	589.92	65.54	5.4	8.3
	GH1	723.89	83.10	732.12	81.34	1.1	2.1
	GH2	780.58	89.09	779.12	86.56	0.18	2.83
	GH3	746.84	85.70	751.31	83.47	0.59	2.60

**Table 6:** (**a**). Flexural values of graphene-filled composites. (**b**) Flexural values of hBN-filled composites.

a			
Fabric	Filler Weight Percentage	Ultimate Flexural Strength (MPa)	Flexural Modulus (GPa)
Kevlar	GK0	158.49	28.33
	GK1	165.56	35.83
	GK2	287.0	66.66
	GK3	253.35	57.56
Hybrid	GH0	369.69	52.49
	GH1	443.82	72.72
	GH2	517.96	99.16
	GH3	480.81	82.89
**b**			
**Fabric**	**Filler Weight Percentage**	**Ultimate Flexural Strength (MPa)**	**Flexural Modulus** **(GPa)**
Kevlar	hBK0	158.49	28.33
	hBK1	202.71	36.66
	hBK2	246.22	41.54
	hBK3	253.35	55.00
Hybrid	hBH0	369.69	52.49
	hBH1	439.91	69.63
	hBH2	457.15	81.10
	hBH3	480.39	97.48

**Table 7:** Micro hardness values of hBN and graphene-filled composites.

Fabric	Filler Weight Percentage	Hardness	Filler Weight Percentage	Hardness
Kevlar	hBK0	159	GK0	159
	hBK1	177	GK1	174
	hBK2	187	GK2	220
	hBK3	200	GK3	186
Hybrid	hBH0	172	GH0	172
	hBH1	176	GH1	191
	hBH2	183	GH2	193
	hBH3	192	GH3	170

**Table 8:** Impact strength values of hBN-filled graphene composites.

Fabric	Filler Weight Percentage	Impact Strength (J/M)	Filler Weight Percentage	Impact Strength (J/M)
Kevlar	GK0	370	hBK0	370
	GK1	520	hBK1	467
	GK2	800	hBK2	598
	GK3	630	hBK3	765
Hybrid	GH0	345	hBH0	345
	GH1	480	hBH1	434
	GH2	650	hBH2	567
	GH3	578	hBH3	716

**Table 9:** Heat deflection values of graphene- and hBN-filled composites.

Fabric	Filler Weight Percentage	Temperature (°C)	Filler Weight Percentage	Temperature (°C)
Kevlar	GK0	80.84	hBK0	80.64
	GK1	84.2	hBK1	85.36
	GK2	91.03	hBK2	87.47
	GK3	85.73	hBK3	89.4
Hybrid	GH0	87.11	hBH0	87.11
	GH1	93.71	hBH1	89.36
	GH2	97.65	hBH2	91.24
	GH3	95.38	hBH3	92.57
